# Supplementary material for: Engineered Pullulan-Collagen-Gold Nano Composite Improves Mesenchymal Stem Cells Neural Differentiation and Inflammatory Regulation
Source: Cells. 2021 Nov 23;10(12):3276. doi: 10.3390/cells10123276 (PMC8699622; doi:10.3390/cells10123276)
Supplement: Supplementary file 1 [file cells-10-03276-s001.zip › cells-1456565-supplementary.pdf]

Engineered Pullulan-Collagen-Gold Nano Composite Improves Mesenchymal Stem Cells Neural Differentiation and Inflammatory Regulation

Supplementary data

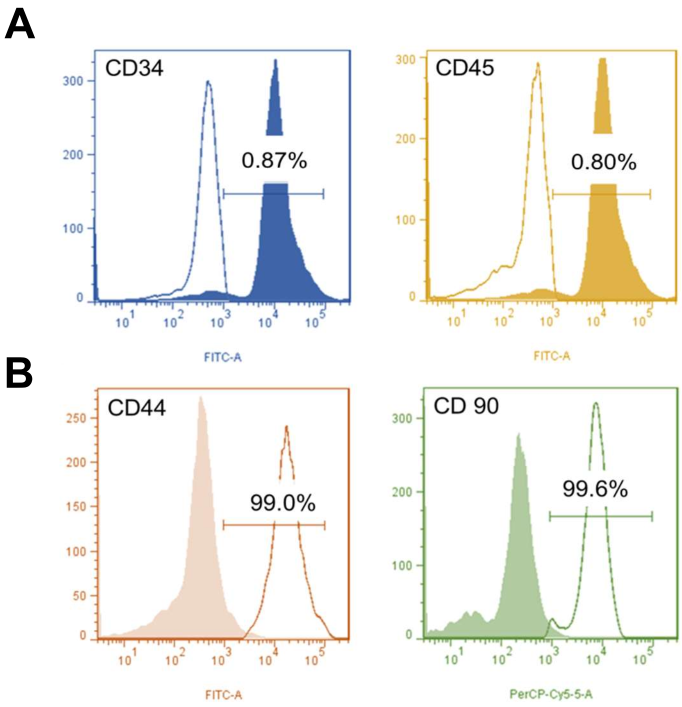

Figure S1. Characterization of MSCs. The antibodies were conjugated with fluorescein isothiocyanate (FITC) or PerCP-Cy5-5-A, the following markers were CD34-FITC, CD45-FITC, CD44-PE, and CD90-PerCP-Cy5-5-A. Further, the specific surface antigens of the MSCs were detected by flow cytometry. **(A)** The quantitative result of CD34 (0.87%) and CD45 (0.80 %), which represented as negative markers. **(B)** The quantitative data of CD44 (99 %) and CD90 (99.6%) expression, which demonstrated as positive markers for the MSCs in current study.

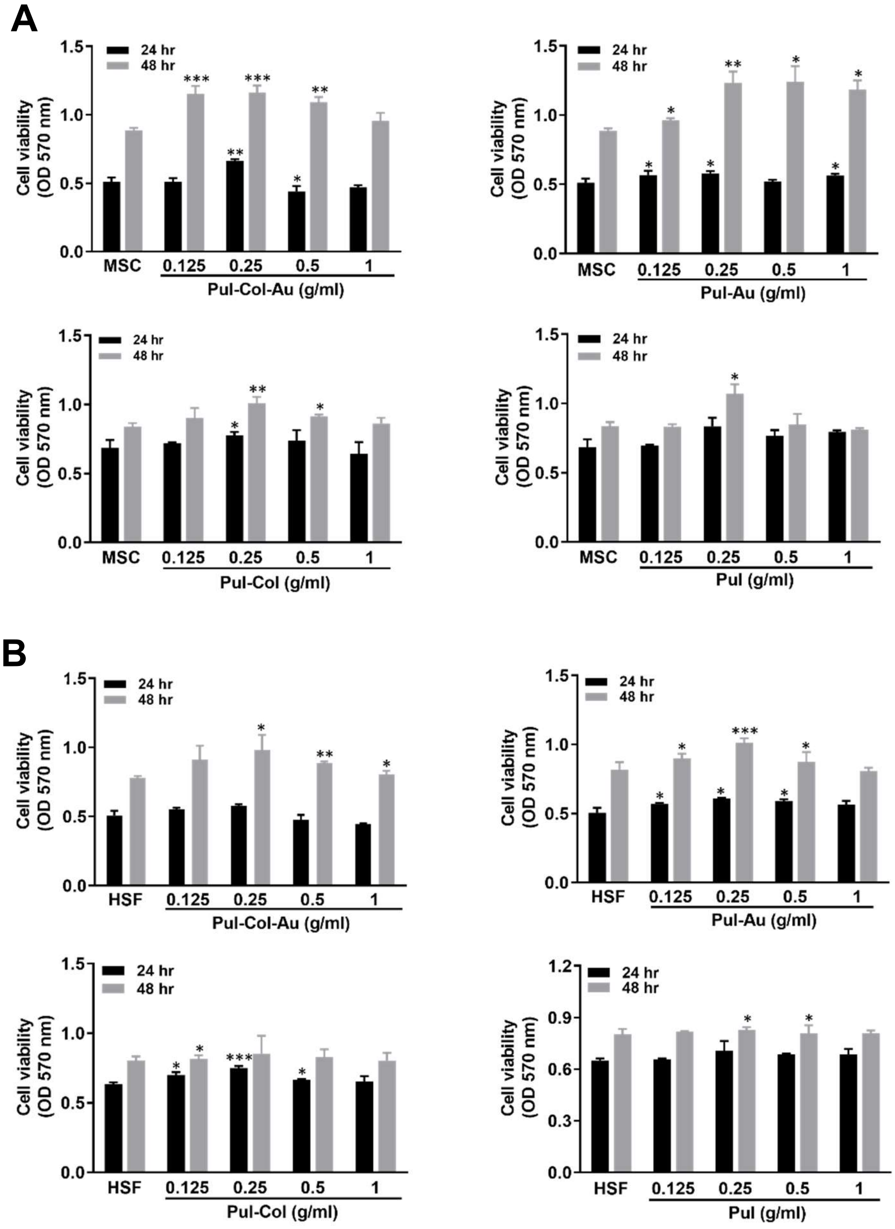

Figure S2. Evaluating the optimal concentration of Pul in each treatment on MSCs and HSFs. The cytotoxicity effect of Pul in various concentrations (0.125, 0.25, 0.5, 1 g/ml) was investigated in Pul-Col-Au, Pul-Au, Pul-Col and Pul treatments. **(A)** The cell viability results indicate that each treatment containing 0.25 g of Pul had the highest value for MSCs (Pul-Col-Au: 1.16, Pul-Au: 1.23, Pul-Col: 1, Pul: 1.07). **(B)** The same results also occurred for HSFs (Pul-Col-Au: 0.98, Pul-Au: 1.01, Pul-Col: 0.85, Pul: 0.82) in each group containing 0.25 g of Pul. Data are represented as mean ± SD of the three independent experiments. *$p < 0.05$, **$p < 0.01$, ***$p < 0.001$: compared to MSC or HSF alone group.

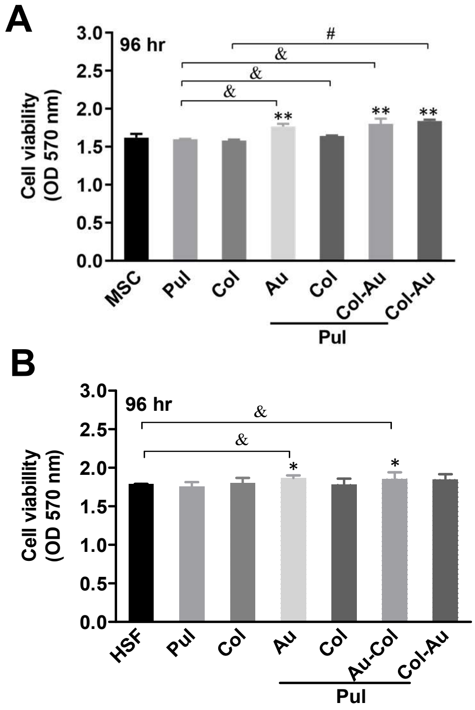

Figure S3. Cell viability of MSCs and HSF s influenced by various materials at 96 hour. **(A-B)** MTT assay was applied to measure the cell viability in long term culture at 96 hours. The above data demonstrated the cell viability in Pul-Col-Au group was significantly higher for both MSCs and HSFs when compared to the control. Data are represented as mean ± SD of the three independent experiments. *p < 0.05, **p < 0.01: compared to MSC or HSF alone group. &p < 0.05: compared to Pul group. #p < 0.05: compared to Col group.

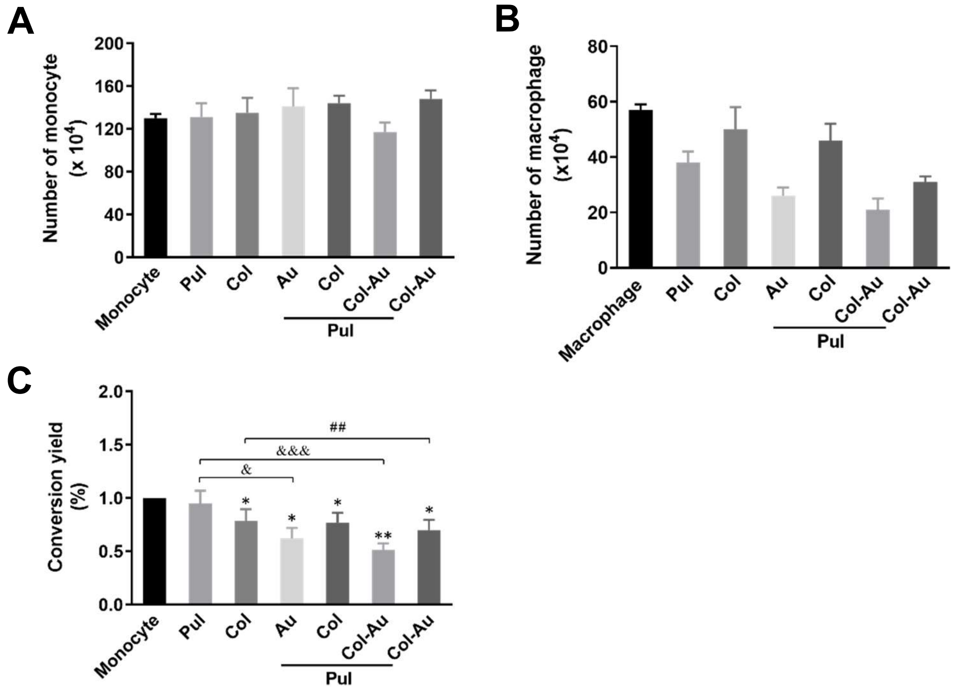

Figure S4. Monocyte conversion yield was investigated at 96 hours. **(A)** The number of monocytes was calculated and demonstrated (Control: 130, Pul: 131, Col: 135, Pul-Au: 141, Pul-Col: 144, Pul-Col-Au: 117, Col-Au: 148). **(B)** The number of macrophages was also calculated (Control: 57, Pul: 38, Col: 50, Pul-Au: 26, Pul-Col: 46, Pul-Col-Au: 21, Col-Au: 31). **(C)** The conversion yield (%) of monocytes to macrophages was further analyzed and displayed (Control: 1, Pul: 0.94, Col: 0.78, Pul-Au: 0.62, Pul-Col: 0.76, Pul-Col-Au: 0.51, Col-Au: 0.69). The results indicate the lowest conversion to be in the Pul-Col-Au group. Data are represented as mean ± SD of the three independent experiments. $^{*}p < 0.05$, $^{**}p < 0.01$,: compared to monocyte alone group. $^{\&}p < 0.05$, $^{\&\&\&}p < 0.001$: compared to Pul group. $^{\#\#}p < 0.01$: compared to Col group.

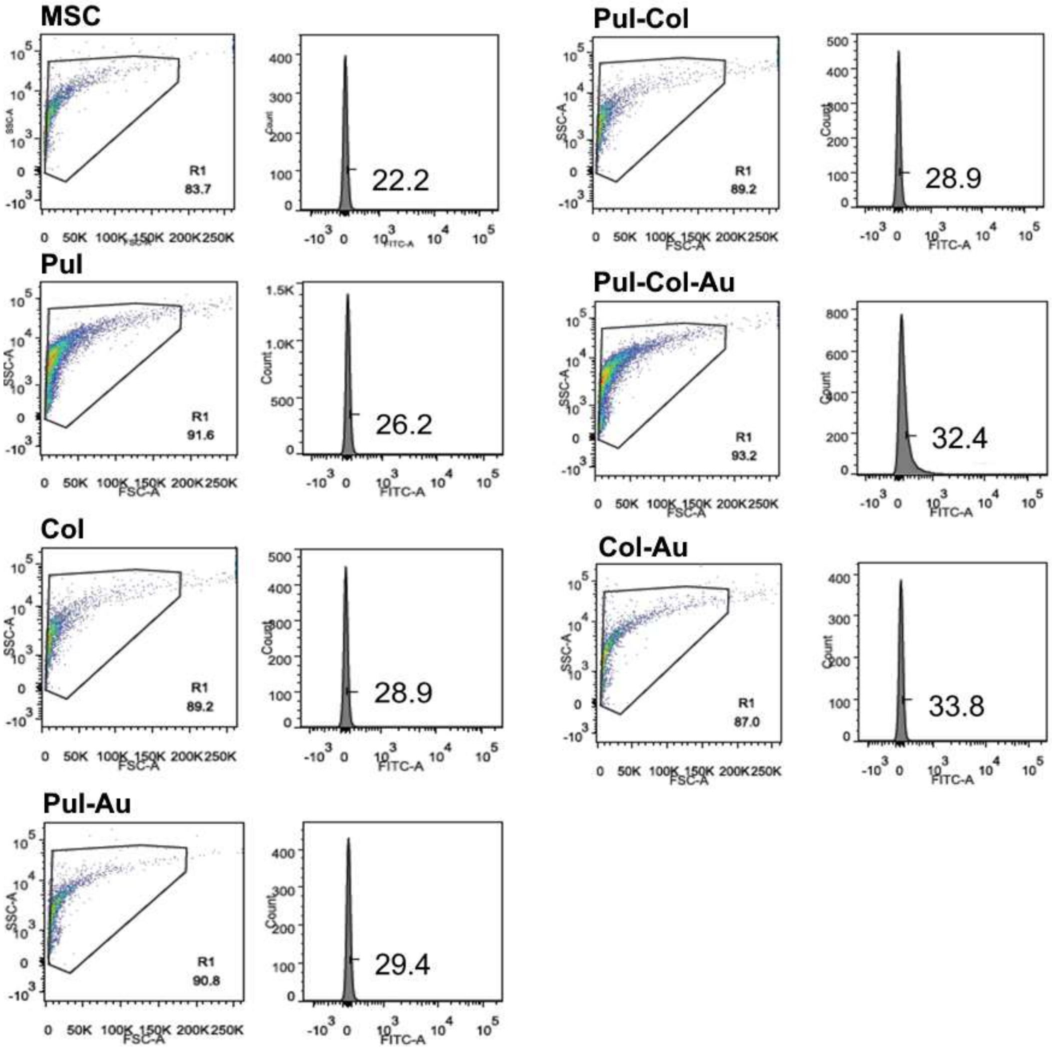

Figure S5. Flow gating results for CXCR4 positive cells detected by flow cytometry. The scatter plots in left panel for each treatment group indicated the gated population of MSCs. Furthermore, the right panel for each treatment groups demonstrated the mean fluorescence of CXCR4 positive MSCs. Subsequently, the fluorescence values of each treatment group were compared relatively to the MSC alone group (represented as the control).

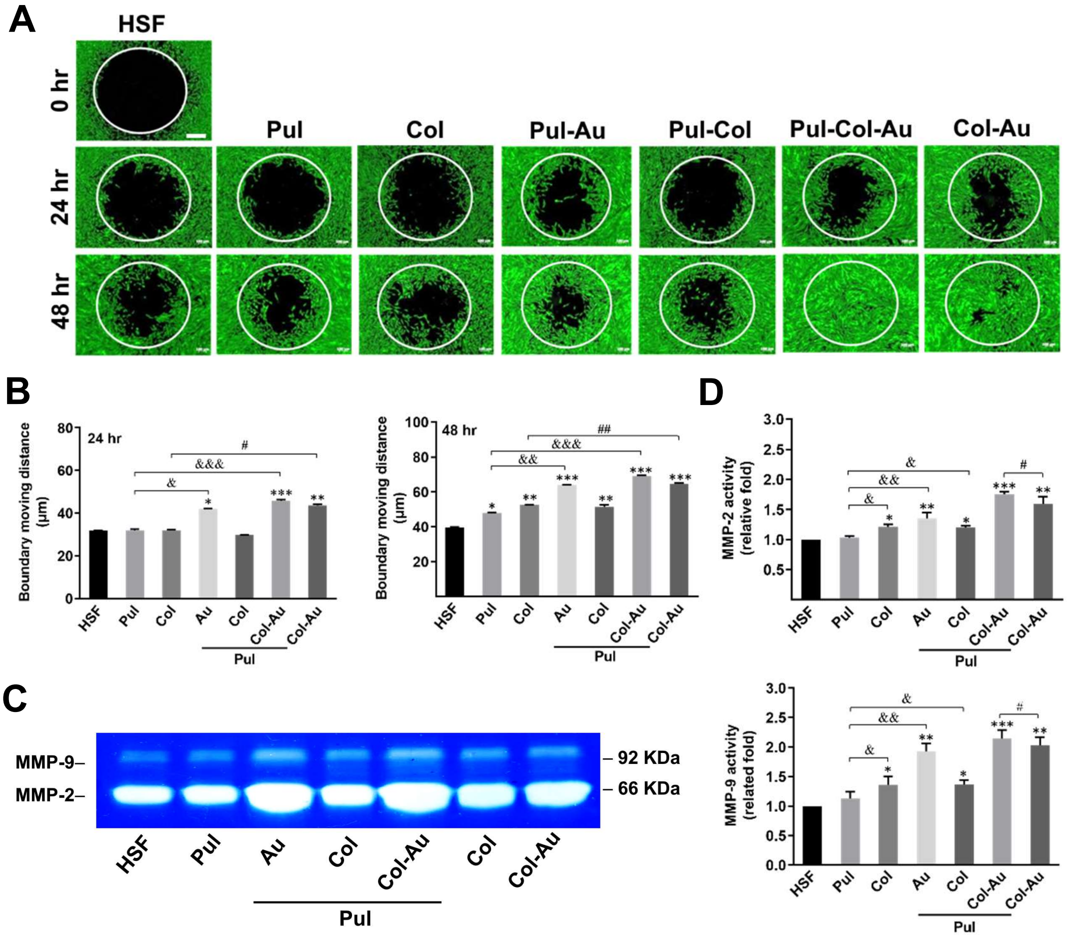

Figure S6. Assessment of biological function for HSFs culturing with various nanomaterials. **(A)** The real-time images of HSFs migration culturing on different nanomaterials were stained with Calcein-AM at 0 (as a reference), 24, and 48 hours. **(B)** The boundary moving distance for each treated group was quantified, with the results also indicating that the highest migration distance was in the Pul-Col-Au group (24 hours: 45.7 µm, 48 hours: 69 µm). $^{*}p < 0.05$, $^{**}p < 0.01$, $^{***}p < 0.001$: compared to HSF alone group. $^{\&}p < 0.05$, $^{\&\&}p < 0.01$, $^{\&\&\&}p < 0.001$: compared to Pul group. $^{\#}p < 0.05$, $^{\#\#}p < 0.01$: compared to Col group. **(C)** The zymogram of MMP activities for HSFs by gelatin zymography analysis at 48 hours. **(D)** The semi-quantification of MMP-2/9 expression was evaluated through Image Pro Plus 5.0 software. The results also elucidate that the greatest expression was in the Pul-Col-Au group (MMP-2: ~ 1.75 fold, MMP-9: ~ 2.14 fold). Data are displayed as mean ± SD of the three independent experiments. $^{*}p < 0.05$, $^{**}p < 0.01$, $^{***}p < 0.001$: compared to HSF alone group. $^{\&}p < 0.05$, $^{\&\&}p < 0.01$: compared to Pul group. $^{\#}p < 0.05$: compared to Pul-Col-Au group.

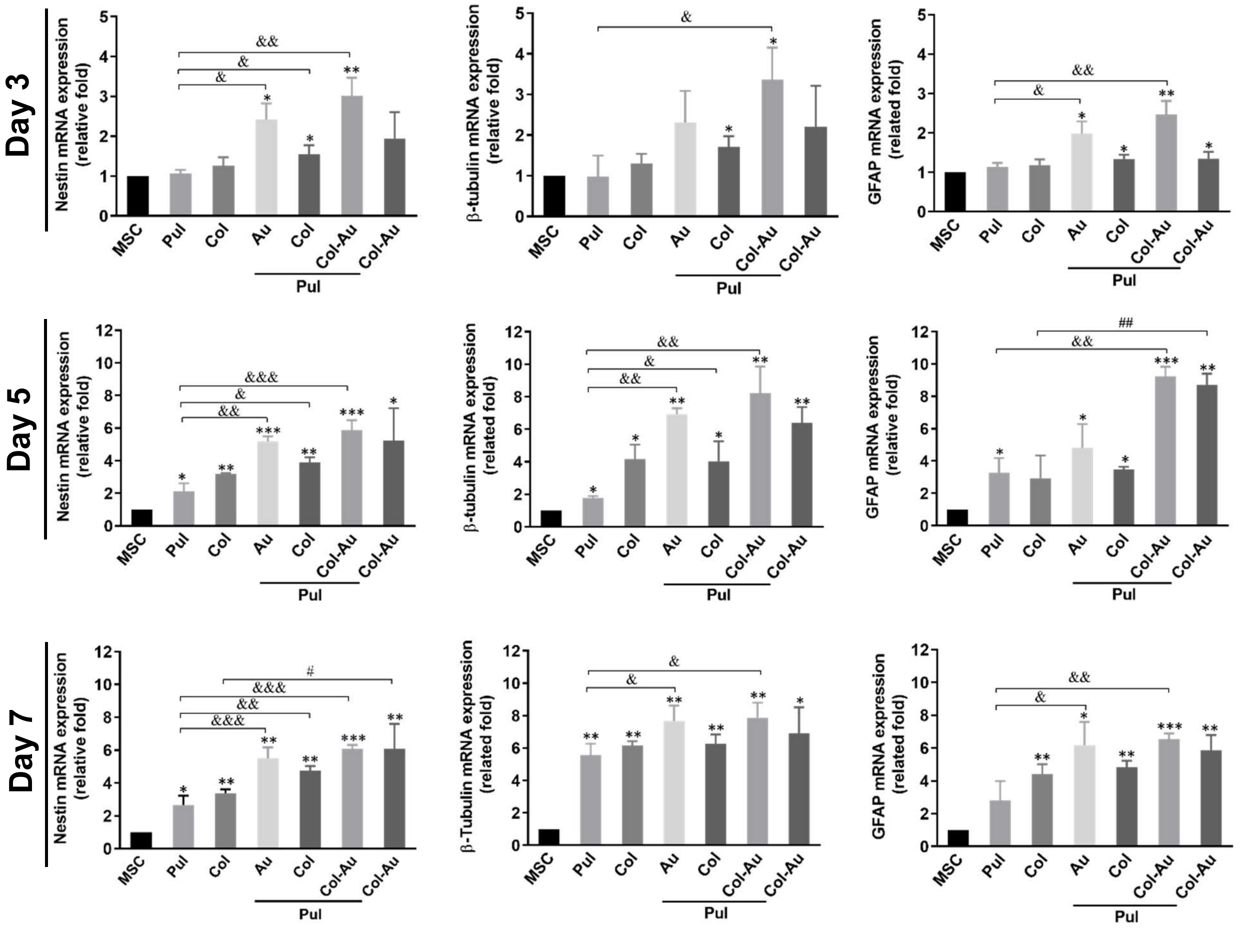

Figure S7. The mRNA expression of neuronal markers in MSCs was evaluated by Real-time PCR assay at days 3, 5, and 7. The results indicate that Pul-Col-Au could induce the highest expression of each neuronal differentiation protein. At day 3, the expression of Nestin, β-tubulin, GFAP was ~ 3.01, ~ 3.36, ~ 2.46 fold respectively. At day 5, it was ~ 5.89, ~ 8.22, ~ 9.23 fold, respectively and at day 7 it was ~ 6.09, ~ 7.86, ~ 6.54 fold, respectively. The quantitative results indicate that Pul-Col-Au had the highest efficiency to induce neurogenic differentiation in MSCs. Data are exhibited as mean ± SD of the three independent experiments. $^{*}p < 0.05$, $^{**}p < 0.01$, $^{***}p < 0.001$: compared to MSC alone group. $^{\&}p < 0.05$, $^{\&\&}p < 0.01$, $^{\&\&\&}p < 0.001$: compared to Pul group. $^{\#}p < 0.05$, $^{\#\#}p < 0.01$: compared to Col group.
